# Can lies be detected unconsciously?

**DOI:** 10.3389/fpsyg.2015.01221

**Published:** 2015-08-25

**Authors:** Wen Ying Moi, David R. Shanks

**Affiliations:** Division of Psychology and Language Sciences, University College LondonLondon, UK

**Keywords:** lie detection, deliberation-without-attention, unconscious thought, decision making

## Abstract

People are typically poor at telling apart truthful and deceptive statements. Based on the Unconscious Thought Theory, it has been suggested that poor lie detection arises from the intrinsic limitations of conscious thinking and can be improved by facilitating the contribution of unconscious thought (UT). In support of this hypothesis, Reinhard et al. (2013) observed improved lie detection among participants engaging in UT. The present study aimed to replicate this UT advantage using a similar experimental procedure but with an important improvement in a key control condition. Specifically, participants judged the truthfulness of eight video recordings in three thinking modes: immediately after watching them or after a period of unconscious or conscious deliberation. Results from two experiments (combined *N* = 226) failed to reveal a significant difference in lie detection accuracy between the thinking modes, even after efforts were made to facilitate the occurrence of an UT advantage in Experiment 2. The results imply that the UT advantage in deception detection is not a robust phenomenon.

## Introduction

Humans regularly encounter and generate deception in their everyday social interactions, even though the act of lying is perceived as morally wrong by a society that values trustworthiness. To place the ubiquity of this phenomenon in perspective, DePaulo et al. (1996) conducted an experiment where college students and community members recorded all their social interactions and all of the lies they told during those interactions every day for a week. A lie was said to occur whenever participants intentionally tried to mislead another individual. By the end of the week, all participants recorded a total of 1,535 lies. For college students, this amounted to two lies per day or one lie in every three social interactions. For community members, this meant one lie per day or one lie in every five social interactions. These findings led DePaulo et al. (1996, p. 991) to conclude that “lying is a fact of daily life." Results from other studies investigating the frequency of deception occurrences (e.g., DePaulo and Kashy, 1998; Weiss and Feldman, 2006) also lend credibility to such a conclusion.

In spite of the pervasiveness of deception, humans are typically poor at discerning between truthful and deceptive statements. We are generally inclined to believe in others and are more adept at identifying truths as being non-deceptive than lies as being deceptive (Bond and DePaulo, 2006). Indeed, Vrij (2008) placed the average person’s lie detection accuracy at about 55%, a level only slightly above chance at 50%. Moreover, in a meta-analysis of 108 studies on deception detection, Aamodt and Custer (2006) found that an individual’s deception detection ability could not be predicted using any the following factors: confidence, age, experience, education, and sex. Those whose occupations dictate regular encounters with deception (e.g., police officers, detectives, and judges) were also no better at detecting it compared to ordinary individuals. The implications of these findings are worrying. Even though an inability to detect lies usually leads to relatively minor consequences in day-to-day, low-stake deceptions, repercussions become much more severe in high-stake contexts such as law enforcement, where individual and societal wellbeing are highly dependent on the ability of law enforcers to form accurate truthfulness judgments.

Hence, it should come as no surprise that much research has been conducted with the aim of improving the deception detection ability of laypeople and law enforcers alike. Research has, for instance, analyzed cues to deception (e.g., Granhag and Strömwall, 2002; DePaulo et al., 2003), investigated people’s beliefs about deception (e.g., Zuckerman et al., 1981; Vrij and Semin, 1996), and devised interview/interrogation methods that facilitate deception detection (e.g., Vrij et al., 2006). The present article explores another possibility, namely that unconscious thought (UT) processes, as characterized in Dijksterhuis and Nordgren’s (2006) Unconscious Thought Theory (UTT), might facilitate improved detection of deception.

Several factors might contribute to people’s poor deception detection performance. One factor is the limit on cognitive processing capacity. Substantial cognitive resources are necessary as deception detection typically requires integrating many partial cues to deception (Vrij, 2008). Another reason is the reliance on misleading cues when evaluating truthfulness as a result of false beliefs about cue validities. Akehurst et al. (1996), for example, found only a weak correlation between beliefs about truthful/deceptive behavior and observations of actual truthful/deceptive behavior. Participants believed that cues such as faltering speech would decrease in frequency during deception but they in fact increased. Gaze aversion, another cue that is universally believed to be associated with deception (Global Deception Research Team, 2006) was also not predictive of actual deceptive behavior. As an individual’s false beliefs often correlate highly with their truthfulness judgments (Bond et al., 1985), it is unsurprising that humans are weak at detecting deception. Although lie detection is typically poor, some cues are objectively diagnostic of deception (DePaulo et al., 2003; Sporer and Schwandt, 2007). For example, truth tellers refer more often to their mental status, and show fewer postural shifts and more facial pleasantness.

All in all, these findings offer a rather bleak outlook on people’s ability to detect deception accurately. In an effort to explore solutions for this problem, the present research examines the potential influences of processes outside conscious awareness on thinking, perception, and behavior (see Bargh, 2011, for a review).

### Unconscious Thought Theory: Background and Critique

Based on findings from a series of decision making experiments, Dijksterhuis (2004), Dijksterhuis and Nordgren (2006), and Dijksterhuis et al. (2006) made the counterintuitive proposal that in forming complex, multi-attribute decisions, engaging in UT would lead to better choices compared to conscious thought (CT). In the original procedure developed by Dijksterhuis and colleagues to elicit what was coined the unconscious thought effect (UTE), participants were given the descriptions of four choice options (e.g., cars) along with 12 feature attributes (e.g., mileage, service, legroom) for each. Each attribute had either a positive or negative valence, and participants were required to choose the best option which was defined as the one that had the most positive attributes. Participants were then assigned to one of the three experimental conditions. In the UT condition, they were instructed to complete some simple tasks for a brief period of time, which meant that their attention was directed away from the attributes and they were distracted from consciously thinking about the options. In the CT condition, participants were asked to think about the options for the same duration, without being given the information about the valence of the options. In the immediate decision (ID) condition, participants made their decisions immediately after seeing all the attributes of each option.

In a series of studies using similar methodologies, Dijksterhuis et al. (2006) found that around 25% of the participants in the CT condition chose the best option, whereas around 60% did so in the UT condition. Dijksterhuis et al. (2006) also varied whether the judgment task was simple or complex (i.e., multiple relevant features to consider; alternatives varying on each of these features).

Subsequently the UTE has been successfully replicated in several studies. In Strick et al.’s (2011) meta-analysis, they found an aggregate effect size of *g* = 0.224 [95% CI: 0.145, 0.303] across 92 studies, a result which they interpreted as offering strong support for the UTE. On the other hand, considerable evidence against the UTE has also been reported. For example, in a recent large scale replication study (*N* = 399) by Nieuwenstein et al. (2015), no significant UTE was observed. In stark contrast to Strick et al.’s (2011) findings, a meta-analysis of 61 UT studies conducted by the same authors (employing a stricter set of inclusion criteria) revealed that the published literature on the UTE includes predominantly non-significant effects (*N* = 45). Furthermore, studies which found a significant UTE (*N* = 12) typically had low sample sizes and were underpowered. The meta-analytic effect size was *g* = 0.018 [-0.10, 0.14], corrected for the high degree of publication bias that was evident. Similarly, Vadillo et al. (2015) found no overall UTE in a meta-analysis focusing solely on studies conducted with medical experts. Thus, the existence of the UTE remains heavily debated and controversial.

The UTE is accounted for by Dijksterhuis and Nordgren’s (2006) UTT. The UTT assumes that conscious but not UT is limited by low cognitive processing capacity. Furthermore, engaging in UT can yield a holistic judgment based on all the attributes of an option, compared to CT which forms judgments based on one or two specific attributes. Finally, the weighting principle states that UT appropriately weighs each attribute based on its relative importance while CT leads to suboptimal weighting.

Despite questions surrounding the UTE’s existence and several weaknesses in its formulation (e.g., González-Vallejo et al., 2008), attempts to replicate the UTE in underexplored settings – such as deception detection – remain worthwhile. Given that humans encounter deception on a daily basis, the findings of such replications will serve to strengthen the ecological validity of evidence supporting or challenging the existence of the UTE. More importantly, replications of the UTE in a deception detection setting would lend support to the hypothesis that humans’ poor deception detection performance is specifically caused by the constraints of CT. Indeed, based on the UTT, the notion that UT can facilitate lie detection seems plausible as characteristics assumed to be present in UT but absent in CT might account for humans’ poor deception detection accuracy. For example, it seems plausible that lie detection is achieved better by an unconscious system that is able holistically to integrate and optimally weight many partially predictive cues in a bottom–up manner than by a conscious system which selects and sub-optimally weights a small number of cues in a top–down, expectancy- or schema-driven way.

Based on these ideas, Reinhard et al. (2013) devised five experiments to test the hypothesis that lie detection by participants in an UT condition would be significantly more accurate compared to CT and standard control conditions. In their Experiment 1, participants watched video recordings of persons recounting a real/false internship experience. Prior to watching the videos, standard control participants learnt of the possibility of deception while UT and CT participants were merely instructed to form impressions. After watching the videos, standard control participants immediately judged each recording’s truthfulness while UT and CT participants learnt of the possibility of deception. For the next 3 min, UT participants completed a non-word search puzzle that served to distract them from consciously thinking about the recordings, while CT participants actively deliberated about each recording’s truthfulness. Finally, both groups made truthfulness judgments. Lie detection accuracy was found to be significantly higher for UT participants compared to CT and standard control participants.

The same trend was observed in Reinhard et al.’s (2013) Experiments 2–4, which had similar procedures to Experiment 1 aside from minor variations in methodology, materials, and conditions included. On the other hand, their Experiment 5 sought to analyze the different cues which UT, CT, and standard control participants relied upon when making truthfulness judgments. They hypothesized that UT participants will rely on more valid cues and integrate all cues in a less stereotypically biased manner. Results confirmed this hypothesis, as UT participants were found to consider five cues when making truthfulness judgments (i.e., postural shifts, facial pleasantness, fidgeting, vocal tension, and unfilled pauses), of which four were objectively diagnostic of deception detection. In contrast, CT and standard control participants relied on fewer cues (i.e., two each) and none of those cues was diagnostic of a message’s truthfulness. In summary, findings from all five experiments conducted by Reinhard et al. (2013) provided strong support for the existence of an UT advantage in deception detection.

As Reinhard et al.’s (2013) research was the first (and to date only) study to discover an UT advantage in a deception detection context, the present study aims to replicate their basic experimental paradigm (i.e., Experiment 1). In addition, the present experiments sought to improve upon the procedure by addressing an important methodological concern. Specifically, the fact that standard control participants – but not those in the UT or CT conditions – were informed about the possibility of deception prior to watching the videos could have influenced their lie detection performance. Hence, differences in judgment accuracies between this condition and the UT and CT conditions might not be attributable to the effects of different thinking modes alone. Put differently, the performance advantage obtained in the UT condition is weakened if one omits findings from the standard control condition and focuses solely on analyzing the pairwise comparison between the UT and CT conditions. This is because the UT condition’s superiority over the CT condition could be caused by CT impairing performance rather than UT improving it (Shanks, 2006). This issue is resolved in the current experiments by treating all three conditions identically up to the deliberation stage. That is, we replace the standard control with an ID condition where participants make truthfulness judgments immediately after seeing the recordings but do not possess foreknowledge of deception. It is hypothesized that UT will still lead to more accurate lie detection compared to the CT and ID conditions, while performance in the latter two will not significantly differ.

The first experiment constitutes an initial attempt to replicate Reinhard et al.’s (2013) Experiment 1, using an improved method as described above. Experiment 2 addresses an issue emerging from the findings of Experiment 1.

## Experiment 1

### Method

#### Participants and Design

The effect sizes observed by Reinhard et al. (2013) for the CT/UT contrast varied between Cohen’s *d*_s_ = 0.56 (Experiment 2) and *d*_s_ = 0.96 (Experiment 3), representing medium to large effects. In order to achieve power of 0.80, sample sizes of 34 per group are required to detect an effect midway between these extremes, *d*_s_ = 0.70. Therefore 116 participants (60 females) aged from 18 to 52 years old (*M* = 25.14, SD = 7.82) were included in the experiment and randomly assigned to one of three conditions: UT (*N* = 37), CT (*N* = 40), and ID (*N* = 39). Participants were recruited from two sources: Prolific Academic, an online crowdsourcing platform (*N* = 89), and the University College London (UCL) Psychology subject pool (*N* = 27). All participants recruited from Prolific Academic and 17 recruited from the UCL Psychology subject pool were paid £3 for the 20 min study, while the remaining subject pool participants received course credit for their participation. All participants listed English as their first language, and provided their informed consent before testing commenced.

#### Apparatus and Stimuli

The experimental survey was designed and run using Qualtrics online survey software. The survey comprised an information acquisition section, deliberation sections for UT and CT participants, and a decision making section.

Experimental stimuli for the information acquisition section consisted of videos of actors telling the truth or lying. Eight individuals (four females) whose first language was English were recruited to pose as actors in these videos in exchange for £3 payment. They were filmed with a digital video camera while recounting one true and one fictitious vacation experience. The filming of truthful responses always preceded deceptive responses, so that actors could reflect upon elements of their truthful response to help them generate their deceptive response as convincingly as possible. Each actor was given 5 min to prepare their true and fictitious stories based on three questions: (1) When, where, and with whom did you go on this vacation? (2) What exactly did you do on this vacation? (3) What did you like/dislike about this vacation? Each of the resulting 16 recordings (*M* = 96.50 s, SD = 7.86 s) showed an actor sitting against a plain wall with his/her face and upper body visible. Average video length did not differ significantly between the truthful (*M* = 94.50 s, SD = 8.96 s) and deceptive responses (*M* = 98.50 s, SD = 6.55 s), *t*(14) = 1.02, *p* = 0.325. Two sets of eight videos were created from the 16 recordings, such that each actor appeared once in each set with a truthful or deceptive response. Each set contained four truthful (two females) and four deceptive (two females) responses. All videos were uploaded onto YouTube before being embedded into the experimental survey. A word search puzzle comprising a 20 × 20 matrix of letters was used to distract UT participants during the deliberation phase.

#### Procedure

The experiment was introduced as “an experiment about interpersonal impression formation.” Participants checked that their computer’s video and audio capabilities were functioning by watching a 5 s video excerpt. This video excerpt had identical settings to the experimental videos, except that the actor was not featured in any of those videos. Participants were also requested to complete the experiment in a quiet environment with minimal distractions.

Subsequently, participants entered the information acquisition phase and were informed that they would be watching eight videos consecutively. Each of these videos was said to consist of an individual describing a past vacation of theirs for ∼90 s. Participants were told to watch each video only once and to repeat it only if they missed parts of it. Their task was to form an impression of what was seen and heard in each video, with no further information given at this point. Participants were then randomly assigned to watch either set of eight videos, each with a predetermined viewing order (see Supplementary Material Appendix A1 for the order and truthfulness of each actor in each set).

For each page, the video’s sequential position and its actor’s fictional name were displayed at the top (e.g., Video 1 of 8: Joe’s Vacation). The video itself was displayed at the center of the page with the dimensions 840 × 472 pixels. Each video was equipped with an auto-play function that enabled it to play immediately after the page was loaded. To ensure that participants watched all the videos completely, all YouTube video controls, keyboard controls, and display information were disabled except for the pause function. Furthermore, participants were prevented from navigating away from the page before each video ended as the “proceed” button for each page was programmed to only appear after the video finished playing.

After watching all eight videos, participants were informed that some of the people they had watched were describing a real vacation experience while others were describing a fictitious vacation. The number of actors who told the truth or lied was not disclosed. Participants were then randomly assigned to the UT, CT, or ID conditions. UT and CT participants underwent a 3 min deliberation phase, where the former were instructed to work on a word search puzzle while the latter were told to actively deliberate about the truthfulness of each actor’s statement. Before commencing this phase, participants in both conditions were informed that they would be making truthfulness judgments on each actor’s statement in the decision making phase at the end of the 3 min. Participants in the ID condition proceeded straight to the decision making phase.

In the decision making test, participants were directed to a page where photographs of all eight actors were listed vertically in the order in which their videos had been presented. Participants judged the truthfulness of each actor’s statement by clicking on the “truth” or “lie” button beneath each actor’s picture. Upon completion, participants were debriefed and thanked.

### Results

Our data analyses are similar to those employed by Reinhard et al. (2013). The hypothesis that participants would make more accurate judgments in the UT than in the CT and ID conditions was tested using signal detection theory. This theory allows for the separation of two measures based on combined information from two parameter estimates: *d*′ (d prime), which in this study measures the ability of participants to make accurate veracity judgments, and *c* (criterion), which measures the general tendency for participants to make truthful or deceptive judgments. Each participant’s discrimination ability (*d*′) was calculated using the loglinear approach to correct hit and false alarm rates to avoid having *z*-scores that were -∞ or +∞ (which results from hit and/or false alarm rates being 0 or 1, respectively). The hit rate is defined as the probability of truthful judgments for truthful videos while the false alarm rate is defined as the probability of truthful judgments for deceptive videos. This approach involves adding 0.5 to both the number of hits and false alarms and adding 1 to the total number of trials before calculating the hit and false alarm rates (Hautus, 1995).

Previous studies have speculated about potential gender differences in the ability to detect deception (cf. Aamodt and Custer, 2006). Moreover, several studies (see Nieuwenstein et al., 2015) revealed that females were more likely to select the best choice options in UTE experiments compared to males. Hence gender is included in the data analysis. All statistical analyses reported in this article were computed in JASP (Love et al., 2015), and the data for this and the following experiment are available at https://osf.io/3qm89.

**FIGURE 1:**
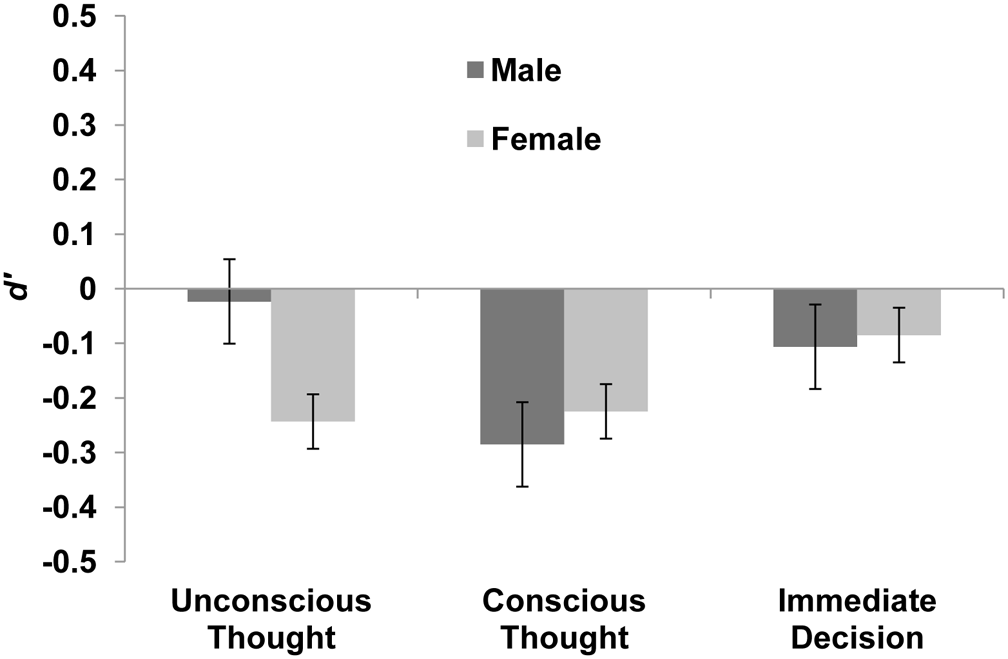
**Mean *d*′ of males and females in each condition of Experiment 1 with +1/-1 SE bars**.

Data from the two sets of videos were combined. Subsequently, discrimination *d*′ was assessed using a 3 (thinking mode) × 2 (gender) between subjects analysis of variance (ANOVA). The main effect of thinking mode was not significant, *F*(2,110) = 0.43, *p* = 0.649, $\eta_{p}^{2}$ = 0.008. The pattern of numerical means revealed that ID participants formed the most accurate truthfulness judgments, *M* = -0.10, 95% CI [-0.35, 0.16], followed by UT participants, *M* = -0.13 [-0.39, 0.13], and CT participants, *M* = -0.26 [-0.50, -0.01]. Neither the main effect of gender, *F*(1,110) = 0.10, *p* = 0.756, $\eta_{p}^{2}$ = 0.001, nor the interaction between gender and mode, *F*(2,110) = 0.35, *p* = 0.708, $\eta_{p}^{2}$ = 0.006, was significant. **Figure 1** illustrates these findings. Across groups and genders, discrimination was significantly worse than chance, *M* = -0.16 [-0.30, -0.02].

Response bias *c* was calculated for each condition. Negative *c* values signify a bias toward judging videos as truthful while positive values signify a bias toward judging them as deceptive. The mean response bias across all conditions was significantly different from zero in the direction of truthful judgments, *M* = -0.16 [-0.23, -0.09]. *c* was then subjected to the same ANOVA as *d*′. The main effect of thinking mode was found to be significant, *F*(2,110) = 5.74, *p* = 0.004, $\eta_{p}^{2}$ = 0.094. The main effect of gender, *F*(1,110) = 0.77, *p* = 0.381, $\eta_{p}^{2}$ = 0.007, and the interaction effect between thinking mode and gender, *F*(2,110) = 0.71, *p* = 0.495, $\eta_{p}^{2}$ = 0.013, were both non-significant. Collapsing across gender, ID participants were most biased toward forming truthful judgments, *M* = -0.31 [-0.43, -0.20], followed by UT participants, *M* = -0.16 [-0.28, -0.04], and finally CT participants, *M* = -0.03 [-0.15, 0.08]. From the confidence intervals it can be seen that the response bias of ID and CT participants differed significantly at *p* < 0.05, while the response bias of UT participants fell somewhere in the middle and did not significantly differ from either the ID or CT condition.

We also computed classification accuracies (percent correct), which are summarized in Table B1 (Supplementary Material). Overall classification accuracy was 46.6% [43.4, 49.7]. Classification accuracy for truthful videos, *M* = 53.9% [49.6, 58.2], was not significantly above chance while accuracy for deceptive videos, *M* = 39.2% [35.0, 43.5], was significantly below chance.

To evaluate the possibility that UT might yield a lie detection benefit over CT for some specific actors, classification accuracies (percent correct) for each actor’s videos across conditions were calculated. Table C1 (Supplementary Material) summarizes the results. For example, of 19 participants who saw Kevin’s truthful video in the UT condition, 12 correctly judged it truthful (hits), while of 18 (different) participants who saw his deceptive video in this condition, 10 judged it truthful (false alarms), for an overall accuracy score of 54.1%.

The table reveals that three actors’ videos could be accurately judged at better than chance (50%) level, and of these two were judged more accurately in the UT than the CT condition. However, even for the most extreme case (Kevin) with a 14.1% UT > CT benefit, the effect was not significant, χ^2^ = 1.53, *p* = 0.217. Thus despite the variability across actors, these results are largely in line with the overall results.

The ANOVA test on *d*′ failed to reveal any significant effect of the different thinking modes on lie detection accuracy. However, null hypothesis testing does not permit any estimation of the extent to which the data support this inference (i.e., the null hypothesis) as compared to the alternative hypothesis that UT participants form more accurate truthfulness judgments compared to CT and ID participants. Bayesian analysis is one approach that allows the relative evidential support for the null and alternative hypotheses to be determined (Rouder et al., 2009). This approach involves the calculation of a Bayes factor BF_01_, defined as the ratio of the probability of the data given the null hypothesis to the probability of the data given the alternative hypothesis. A Bayes factor that is greater than 1 indicates that belief should be adjusted in favor of the null hypothesis, while a Bayes factor that is less than 1 indicates that belief should be adjusted in favor of the alternative hypothesis. Comparisons of *d*′ between the UT and CT conditions and between the UT and ID conditions (using the Cauchy distribution, scale *r* on effect size = 1.0) yielded Bayes factors of 4.65 and 5.64, respectively, providing substantial support for the null hypotheses that UT does not increase lie detection in comparison to conscious or immediate decisions.

### Discussion

Experiment 1’s main objective was to replicate Reinhard et al.’s (2013) finding that participants who engaged in unconscious deliberation formed more accurate truthfulness judgments compared to those in CT and standard control (or in this experiment’s case, ID) conditions. However, the experiment found no significant difference in lie detection accuracies among UT, CT, and ID participants. The numerical trend of *d*′ indicated that participants in fact performed most accurately in the ID condition, followed by the UT and finally CT conditions. This contradicts the UTE pattern found by Reinhard et al. (2013) and previous decision making studies. Computation of Bayes factors also revealed that a rational observer who considers the alternative hypothesis against the null hypothesis should, given these results, adjust his/her belief in favor of the null hypothesis by a factor of around 5. Indeed, Experiment 1’s results suggest that there is no benefit for any form of deliberation during lie detection.

Analysis of participants’ overall classification accuracy revealed that they were able to discriminate between truthful and deceptive videos, but were classifying them incorrectly. Specifically, participants were discriminating the truthful videos at chance level but were significantly more likely to erroneously classify deceptive videos as truthful. One potential account for this is that the actors were very convincing liars. As the actors were encouraged to reflect on their performance in the truthful videos to make their deceptive statements as believable as possible, it is possible that they incorporated cues that are typically associated with truthful responses into their deceptive videos. Previous research (e.g., DePaulo et al., 2003; Sporer and Schwandt, 2007) into the ways in which liars act differently from truth-tellers revealed several cues to be objectively diagnostic for deception detection. For instance, it was found that truth tellers referred more often to their mental status, showed fewer postural shifts and more facial pleasantness. Using postural shifts as an example, it is possible that the actors incorporated fewer postural shifts into their deceptive accounts than they would otherwise have done in an effort to be convincing. As a result of this overcompensation, actors unknowingly enhanced the (erroneous) credibility of their deceptive videos.

## Experiment 2

Given that participants’ overall classification accuracies in Experiment 1 were significantly below chance level, it could be argued that the failure to replicate Reinhard et al.’s (2013) finding was due to the presence of inappropriate cues in the experimental videos that confounded the discrimination between truthful and deceptive responses. Thus, Experiment 2 will once again attempt to replicate the UT advantage found in Reinhard et al.’s (2013) Experiment 1, but this time ensuring that relevant cues are present in truthful and deceptive videos. Specifically, cues that were found by Reinhard et al. (2013) to correlate with UT participants’ truthfulness judgments (i.e., postural shifts, facial pleasantness, fidgeting, and length of unfilled pauses) were explicitly incorporated into the experimental videos.

The rationale for incorporating these cues is twofold. Firstly, given that three out of these four cues are objectively diagnostic of lie detection, their incorporation should allow for the truthful and deceptive videos to be objectively discriminated. If the truthfulness judgments of participants across all conditions are indeed facilitated, this would indicate that participants base their judgments on cues available in the experimental videos. Secondly, this creates a more conducive environment to elicit the UTE, should it be present. If the lie detection accuracy of UT participants was to be facilitated more compared to CT and ID participants, this would lend support to Reinhard et al.’s (2013) finding of an UT advantage in a deception detection context.

### Method

#### Participants and Design

One hundred and ten participants (55 females) aged from 18 to 29 years old (*M* = 22.13, SD = 2.65) were recruited via Prolific Academic. None of the participants had taken part in Experiment 1, and all were paid £3 for the 20 min study. They were randomly assigned to one of three conditions: UT (*N* = 37), CT (*N* = 40), and ID (*N* = 33). Similar to Experiment 1, all participants listed English as their first language, and provided their informed consent before testing commenced.

#### Apparatus and Stimuli

Experiment 2 utilized similar apparatus and stimuli as Experiment 1, aside from the videos used in the information acquisition phase. Eight further individuals (four females) whose first language was English were recruited to pose as actors in the new videos in exchange for £3 payment. The actors were once again filmed with a digital video camera while recounting a true and a fictitious vacation experience. The filming order of responses (i.e., truthful before deceptive) was identical to Experiment 1, as were the timing and three questions provided for the actors to prepare their stories. In addition, actors were instructed to modulate the frequency of four out of the five cues that were found by Reinhard et al. (2013) to correlate with UT participants’ judgments of truthfulness: postural shifts, facial pleasantness, fidgeting, and unfilled pauses. Appendix D (Supplementary Material) details the definitions of these cues as extracted from DePaulo et al. (2003). The fifth cue – less vocal tension – was excluded as it was difficult for actors to control. Similarly, frequency (instead of duration) of unfilled pauses was manipulated as the former was easier for actors to keep track of. Thus, in truthful recordings, actors were instructed to show more fidgeting and facial pleasantness while avoiding frequent postural shifts and silent pauses. In contrast, in deceptive recordings, they were instructed to show more postural shifts and silent pauses while avoiding fidgeting or looking pleasant. All recorded videos were compared with those from Experiment 1 to confirm that the manipulated cues were displayed more/less often.

Each of the resulting 16 recordings (*M* = 101.00 s, SD = 6.19 s) showed an actor sitting against a plain wall with his/her face and upper body visible. The average video length did not differ between the truthful (*M* = 98.25 s, SD = 5.09 s) and deceptive responses (*M* = 103.75 s, SD = 6.23 s), *t*(14) = 1.93, *p* = 0.074, and the average length did not differ significantly between Experiments 1 and 2, *t*(30) = 1.80, *p* = 0.082. Once again, two sets of eight videos, each with four truthful (two females) and four deceptive (two females) responses were created from the 16 recordings. Appendix A2 (Supplementary Material) lists the order in both sets.

#### Procedure

The procedure was identical to that of Experiment 1.

### Results

**FIGURE 2:**
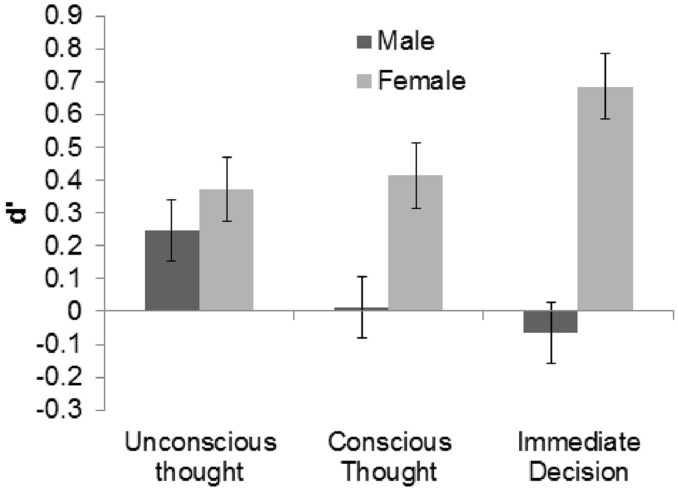
**Mean *d*′ of males and females in each condition of Experiment 2 with +1/-1 SE bars**.

Data from participants who watched the different sets of videos were again combined under their respective thinking modes for analysis. Each participant’s *d*′ was calculated using hit and false alarm rates that were corrected using the loglinear approach. Across groups and genders, and confirming the success of our manipulation of the video materials, discrimination was significantly better than chance, *M* = 0.28, 95% CI [0.09, 0.47], Cohen’s *d*_s_ = 0.28. Discriminability *d*′ was then subjected to a 3 (thinking mode) × 2 (gender) between subjects ANOVA. Similar to Experiment 1, the main effect of thinking mode was not significant, *F*(2,104) = 0.12, *p* = 0.887, $\eta_{p}^{2}$ = 0.002. The numerical pattern of means revealed that ID and UT participants made the most accurate judgments, *M* = 0.31 [-0.03, 0.65] and *M* = 0.31 [-0.02, 0.63], respectively, followed by CT participants, *M* = 0.21 [-0.10, 0.53]. However, the main effect of gender was found to be significant, *F*(1,104) = 5.02, *p* = 0.027, $\eta_{p}^{2}$ = 0.045, although it did not interact with thinking mode, *F*(2,104) = 0.86, *p* = 0.427, $\eta_{p}^{2}$ = 0.015. Specifically, females, *M* = 0.49 [0.22, 0.76], formed more accurate judgments than males, *M* = 0.06 [-0.20, 0.33]. **Figure 2** illustrates these results.

To investigate participants’ response bias, *c* was calculated using corrected hit and false alarm rates. Like Experiment 1, mean response bias across all conditions was found to be significantly different from zero in the direction of truthful judgments, *M* = -0.18 [-0.23, -0.14]. *c* was then evaluated using the same ANOVA as *d*′. The main effect of thinking mode was not significant, *F*(2,104) = 0.08, *p* = 0.927, $\eta_{p}^{2}$ = 0.001. Similarly, the main effect of gender, *F*(1,104) = 0.16, *p* = 0.692, $\eta_{p}^{2}$ = 0.001, and the gender × mode interaction, *F*(2,104) = 1.79, *p* = 0.171, $\eta_{p}^{2}$ = 0.033, were not significant.

Similar to Experiment 1, participants’ classification accuracy (percent correct) was calculated (Appendix B2, Supplementary Material). Overall classification accuracy, *M* = 55.8% [51.7, 59.9], improved compared to Experiment 1 and was significantly greater than chance (50%). Specifically, classification accuracy for truthful videos, *M* = 63.6% [59.4, 67.9], was significantly above chance while classification accuracy for deceptive videos, *M* = 48.0% [43.1, 52.8], was not.

The accuracy of classification of each actor’s truthful and deceptive videos was also examined using a similar method as in Experiment 1 (see Table C2, Supplementary Material). Consistent with the overall improvement compared to Experiment 1, six actors’ videos could be accurately judged at better than chance (50%) level, but only two of these were judged more accurately in the UT than the CT condition. Even for the most extreme case (James) with an 18.9% UT > CT benefit, the difference was not significant, χ^2^ = 2.81, *p* = 0.094.

Once again, a Bayes factor analysis was conducted to estimate the extent to which the data support the null hypothesis. The Bayes factors (BF_01_) for comparisons of *d*′ between the UT and CT conditions and between the UT and ID conditions were 5.25 and 5.50, respectively, again offering substantial support for the hypothesis that UT does not promote lie detection.

### Discussion

Building on Experiment 1’s failure to replicate Reinhard et al.’s (2013) findings, Experiment 2 sought to investigate whether UT leads to enhanced lie-detection accuracy compared to CT and ID, by improving the objective discriminability of the experimental videos and creating an optimal environment to elicit the UTE. However, truthfulness judgments among participants in the three thinking modes still did not differ significantly. Indeed, results from the Bayes factor analysis revealed that the experimental data offered strong support for the null hypothesis. The non-significant trend reflected the same pattern as Experiment 1, with ID participants making the most accurate judgments and CT the least. Hence, the present findings support the notion that the UTE is not a robust phenomenon in deception detection, while also echoing Experiment 1’s suggestion that there is minimal benefit of any form of deliberation in forming deception detection judgments.

Contrary to the findings in Experiment 1, females were found to make significantly more accurate judgments compared to males. One possible account for women’s superiority in this experiment is that they were better than men at detecting the manipulated cues. For instance, Hall (1978) provided some evidence that women tend to be more adept at decoding non-verbal cues than men, especially in visual-plus-auditory situations. Even if this is the case, the present results suggest that gender differences in sensitivity did not vary as a function of the different thinking modes.

Also in contrast to Experiment 1 is the finding that participants were better at discriminating between truthful and deceptive videos. This suggests that the experimental videos employed in the current experiment (unlike those in Experiment 1) incorporated appropriate discriminating cues. Indeed the overall accuracy level (55.8%) is very close to that obtained by Reinhard et al. (2013, Experiment 1), 54.7%. Hence the difference in outcome cannot be attributed to any overall difference in task difficulty (and of course the significant gender effect shows that our study was sensitive to at least one factor). Finally, the degree of response bias displayed by participants was not found to significantly differ as a function of thinking mode. This is consistent with Reinhard et al.’s (2013) findings and lends support to the suggestion that the significant difference found in Experiment 1 was an anomaly.

Note that in both experiments there is some tendency for males to make more accurate judgments in the UT than the CT condition, but even this *post hoc* comparison is not significant. Combining the data from male participants across both experiments, *d*′ did not differ, *t*(76) = 1.09, *p* = 0.28, and the null hypothesis is supported, BF_01_ = 3.64.

## General Discussion

The main aim of this research was to verify whether a UTE exists in the lie detection domain. Specifically, it was hypothesized that engaging in UT would lead to better lie-detection accuracy compared to CT and immediate choice. However, both experiments failed to observe any significant difference in lie detection accuracy across the three thinking modes. On the contrary, Bayes factor analyses found substantial support in favor of the null hypothesis that the different thinking modes do not lead to differences in lie detection accuracy.

Despite largely adhering to the procedure of Reinhard et al.’s (2013) studies, the present experiments failed to replicate their finding that unconscious processing leads to superior deception detection performance. One could argue that the discrepant results in Experiment 1 were due to the presence of confounding cues in the experimental videos, which did not allow for accurate truth/deception discrimination. However, Experiment 2 still did not find any significant advantage for engaging in UT, in spite of the fact that the truthful and deceptive videos in this experiment were objectively discriminable. This finding is especially notable given that the cues incorporated into the videos in Experiment 2 were explicitly intended to facilitate the occurrence of a UTE.

Statistical power is unlikely to be a contributing factor in these failed replications, as a power analysis suggested that our sample sizes (116 and 110 in Experiments 1 and 2, respectively) were adequate to detect the effect obtained by Reinhard et al. (2013), and their Experiment 1 included substantially fewer participants (66). Note also that the Bayesian analyses, which yielded substantial support for the null hypothesis, implicitly take sample size into account.

### Accounting for the Replication Failure

The present findings could be the result of two alternative possibilities: either the UTE found by Reinhard et al. (2013) was a false positive, or engaging in UT truly leads to enhanced lie detection accuracy compared to CT and immediate choice, but minor methodological differences between the present study and Reinhard et al.’s (2013) prevented detection of the effect. We consider four such differences.

First, the length of the video stimuli used across both experiments in the current study (*M* = 99 s) was appreciably shorter than those used in Reinhard et al.’s (2013) Experiment 1 (*M* = 228 s). The rationale behind using shorter videos stemmed from a reluctance to have participants watch videos consecutively for more than half an hour (228 s × 8 videos = 1824 s), which could have induced boredom and inattention. Is it likely that this shorter presentation time could have in some way hampered the occurrence of a UTE? Strick et al.’s (2011) meta-analysis revealed that the UTE tended to be larger when information presentation times were shorter. If anything, a shorter video length should therefore have facilitated the UTE. Moreover in their Experiments 2 and 4 Reinhard et al. (2013) themselves used much shorter videos (30 s) and still obtained UTEs. Nevertheless shorter videos obviously contain fewer cues on which to form a truthfulness judgment.

Secondly, several differences exist between the videos utilized in the present experiments and those used in Reinhard et al.’s (2013) Experiment 1. Reinhard et al.’s (2013) experiment was conducted in German while the present experiments were conducted in English. Actors in Reinhard et al.’s (2013) videos spoke about an internship experience in a realistic setting (i.e., they were dressed in business attire and filmed in a room that looked appropriate for an employment interview), while actors in the present experiments spoke about a vacation experience in a neutral setting (i.e., they were dressed normally and were filmed against a blank wall). In addition, all eight actors in Reinhard et al. (2013) were males while the present experiments used an even mix of male and female actors. These differences could potentially contribute to the failed replication. If we look at lie detection performance separately for the eight male and eight female actors in the present experiments (data in Tables C1 and C2, Supplementary Material), overall detection accuracy is in fact slightly higher for the females. For male actors in Experiment 2, *d*′ is somewhat higher in the UT (*M* = 0.13) compared to the CT (*M* = -0.10) and I (*M* = 0.09) conditions but these differences are far from significant (*p* > 0.3 in both cases). Nevertheless, actor gender should certainly be borne in mind as a potential moderator in future research.

Thirdly, in contrast to Reinhard et al.’s (2013) laboratory based experiments, both experiments in this study were conducted entirely online. The fact that online subjects participate in experiments under self-supervised conditions raises some potential questions about the reliability of the data obtained compared to laboratory based experiments, and the level of engagement of participants throughout the experiment. But a growing literature demonstrates rather similar data patterns in a broad range of laboratory and online experiments, including ones evaluating decision making (e.g., Paolacci et al., 2010; Germine et al., 2012; Klein et al., 2014). This is certainly something to be explored further in future research, but the available evidence provides no strong support for this being an important moderating factor. One of the clear advantages of online experiments is that direct human interaction between researcher and participant is limited thus leaving less room for experimenters to bias participants’ responses.

Finally, the task used to distract UT participants during the deliberation phase in the current study was slightly different from Reinhard et al.’s (2013). Specifically, Reinhard et al. (2013) had UT participants complete a 15 × 15 non-word search puzzle, with an undisclosed number of non-words to be found. The distraction task used in the present experiments was a 20 × 20 word search puzzle, with 18 words to be found. Although usage of different forms of word search puzzle seems like a trivial methodological detail, Strick et al. (2011) did find that the UTE tended to be larger in studies using word search puzzles (as opposed to anagrams or *n-*back tasks) as a distraction task. Given that the nature of a distraction task could lead to different outcomes, it is possible that engaging in a word search puzzle with a different format could lead to a failure in replicating the UTE as well.

Note of course that even if the UT > CT effect observed by Reinhard et al. (2013) is reproducible, they provided no evidence that it demonstrates a benefit of UT: equally possible is that it results from a detriment of CT (Shanks, 2006). Without an appropriate ID control condition, these possibilities cannot be distinguished. The experiments reported here included just such a control condition, and no hint of a benefit of UT was observed.

### Implications and Limitations

Instead of endorsing Reinhard et al.’s (2013) proposed solution to improve humans’ poor deception detection performance, the present findings affirm the pessimistic outlook offered by previous deception detection studies on people’s ability to distinguish truths from lies (e.g., Vrij, 2008), and contribute to existing literature that argues against the existence of a UTE (e.g., Nieuwenstein et al., 2015). While the current results do not speak to potential improvements in deception detection accuracy using other methodologies, they cast some doubt on the notion that deception detection accuracy can be significantly facilitated by engaging in UT. Indeed, the fact that ID participants consistently performed best and CT worst (albeit non-significantly) in both of our experiments suggests that there is no benefit of any form of deliberation when it comes to lie detection.

Whatever the conclusions about UT, it is interesting that conscious, deliberate thought provides such little benefit over immediate thought in the conditions tested here and elsewhere. A possible explanation is that the UTE procedure places excessive burdens on working memory as participants try to retain a considerable amount of highly confusable information prior to making their judgments. Another possibility is that participants treat the task as an online judgment problem, forming their beliefs while observing the videos, rather than subsequently. Newell and Shanks (2014) review several accounts of why CT yields such little benefit in UTE experiments.

One limitation of the present studies is the lack of specific assessment of the cues on which participants based their judgments. Future research should measure self-reported strategies, and also present specific probe items. For example, participants could be shown ‘thin slices’ of behavior in which a single cue is manipulated.

Evidence from other experimental methods is similarly inconclusive about the role of unconscious processes in lie detection. For instance, Albrechtsen et al. (2009) reported better deception detection when participants viewed ‘thin-sliced’ 15 s versions of true and false confessions than longer 3 min versions, and also detected deception better in the longer videos when they viewed them concurrently with performing a secondary *n*-back task. But as with the UT > CT effect in UTE experiments, these findings do not distinguish between a benefit of UT and a detriment of CT. In another example, ten Brinke et al. (2014) presented participants with videos of actors either telling the truth or lying about a theft. Discrimination was at chance under explicit (conscious) conditions similar to those of the present ID condition. In contrast, during implicit (unconscious) tests, participants responded significantly differently to images of truthful and deceptive actors. ten Brinke et al.’s (2014) rationale was to suppress conscious processing and hence permit the capacity-unlimited, optimal-weighting unconscious to operate effectively. In light of their results they concluded that deceptive cues from the actors can be detected and “leak” into unconscious but not conscious decisions. But as pointed out by Levine and Bond (2014), the chance-level discrimination ten Brinke et al. (2014) observed under explicit conditions is anomalous: although lie detection is often poor, meta-analyses (as noted in the Introduction) show that it yields an effect size estimate of around *d* = 0.4, which is greater than the implicit effects observed by ten Brinke et al. (2014). As with the UTE, more research with these methods is needed before firm conclusions can be drawn.

In light of these considerations and the results obtained in our experiments, Reinhard et al.’s (2013) suggestion to incorporate unconscious processes into police training manuals on deception detection seems premature. Furthermore, their claim that “the human mind is not unfit to distinguish between truth and deception but this ability resides in previously overlooked processes” (Reinhard et al., 2013, p. 721) appears too simplistic. More in-depth research into these questions as well as further replications of the UTE in the deception detection context is clearly needed. In the meantime, instead of manipulating one’s thinking mode in the hope of improving lie detection, it is perhaps wise to stick to methods that have been shown in many studies to be effective in facilitating deception detection (Vrij, 2008).

## Conflict of Interest Statement

The authors declare that the research was conducted in the absence of any commercial or financial relationships that could be construed as a potential conflict of interest.
