# Supplementary material for: Can lies be detected unconsciously?
Source: Front Psychol. 2015 Aug 25;6:1221. doi: 10.3389/fpsyg.2015.01221 (PMC4548087; doi:10.3389/fpsyg.2015.01221)
Supplement: Supplementary file 1 [file Data_Sheet_1.DOCX]

**Appendix A**

Table A1.

Position and truthfulness of each actor in each set in Experiment 1.

| Fictional Name of Actor | Gender of Actor | Position Set 1 | Truthfulness | Position Set 2 | Truthfulness |
| --- | --- | --- | --- | --- | --- |
| Joe | M | 1 | D | 4 | T |
| Lucas | M | 2 | T | 7 | D |
| Sarah | F | 3 | D | 2 | T |
| Ruby | F | 4 | T | 8 | D |
| Ella | F | 5 | D | 6 | T |
| Olivia | F | 6 | T | 5 | D |
| Kevin | M | 7 | T | 1 | D |
| William | M | 8 | D | 3 | T |

Note: M = male, F= female, D = deception, T = truthful.

Table A2.

Position and truthfulness of each actor in each set in Experiment 2.

| Fictional Name of Actor | Gender of Actor | Position Set 1 | Truthfulness | Position Set 2 | Truthfulness |
| --- | --- | --- | --- | --- | --- |
| Karen | F | 1 | D | 6 | T |
| Paul | M | 2 | T | 7 | D |
| Kenneth | M | 3 | D | 4 | T |
| Steven | M | 4 | T | 5 | D |
| Patricia | F | 5 | D | 2 | T |
| Michelle | F | 6 | T | 1 | D |
| Nancy | F | 7 | T | 8 | D |
| James | M | 8 | D | 3 | T |

Note: M = male, F= female, D = deception, T = truthful.

**Appendix B**

Table B1.

Mean (*SD*) classification accuracy (% correct) as a function of thinking mode for truthful and deceptive videos in Experiment 1.

Note: UT = unconscious thought; CT = conscious thought; ID = immediate decision.

| Thinking Mode | Truthful (%) | | Deceptive (%) | | Overall (%) | | | |  |
| --- | --- | --- | --- | --- | --- | --- | --- | --- | --- |
|  | *M* | *SD* | *M* | *SD* | | *M* | *SD* | *N* | |
| UT | 54.7 | 23.5 | 40.5 | 20.7 | | 47.6 | 18.4 | 37 | |
| CT | 46.3 | 23.0 | 42.5 | 26.1 | | 44.4 | 18.3 | 40 | |
| ID | 60.9 | 22.1 | 34.6 | 21.9 | | 47.8 | 15.4 | 39 | |
| Overall | 53.9 | 23.5 | 39.2 | 23.1 | | 46.6 | 17.3 | 116 | |

Table B2.

Mean (*SD*) classification accuracy (% correct) as a function of thinking mode for truthful and deceptive videos in Experiment 2.

| Thinking Mode | Truthful (%) | | Deceptive (%) | | Overall (%) | | | |  |
| --- | --- | --- | --- | --- | --- | --- | --- | --- | --- |
|  | *M* | *SD* | *M* | *SD* | | *M* | *SD* | *N* | |
| UT | 64.9 | 21.6 | 48.0 | 21.6 | | 56.4 | 19.5 | 37 | |
| CT | 61.9 | 21.2 | 46.9 | 26.1 | | 54.4 | 20.7 | 40 | |
| ID | 64.4 | 25.0 | 49.2 | 29.6 | | 56.8 | 25.0 | 33 | |
| Overall | 63.6 | 22.4 | 48.0 | 25.6 | | 55.8 | 21.5 | 110 | |

Note: UT = unconscious thought; CT = conscious thought; ID = immediate decision.

**Appendix C**

Table C1.

Mean classification accuracy (% correct) for each actor’s truthful and deceptive videos in each thinking mode in Experiment 1.

| Actor | UT | CT | ID | Mean Accuracy |
| --- | --- | --- | --- | --- |
| Joe | 51.4 | 37.5 | 46.2 | 44.8 |
| Lucas | 56.8 | 60.0 | 43.6 | 53.4 |
| Sarah | 35.1 | 45.0 | 59.0 | 46.6 |
| Ruby | 54.1 | 52.5 | 51.3 | 52.6 |
| Ella | 45.9 | 42.5 | 41.0 | 43.1 |
| Olivia | 32.4 | 40.0 | 38.5 | 37.1 |
| Kevin | 54.1 | 40.0 | 59.0 | 50.9 |
| William | 51.4 | 40.0 | 43.6 | 44.8 |

Note: UT = unconscious thought; CT = conscious thought; ID = immediate decision.

Table C2.

Mean classification accuracy (% correct) for each actor’s truthful and deceptive videos in each thinking mode in Experiment 2.

| Actor | UT | CT | ID | Mean Accuracy |
| --- | --- | --- | --- | --- |
| Karen | 51.4 | 57.5 | 60.6 | 56.4 |
| Paul | 56.8 | 60.0 | 57.6 | 58.2 |
| Kenneth | 48.6 | 52.5 | 57.6 | 52.7 |
| Steven | 56.8 | 45.0 | 45.5 | 49.1 |
| Patricia | 73.0 | 62.5 | 69.7 | 68.2 |
| Michelle | 48.6 | 65.0 | 51.5 | 55.5 |
| Nancy | 64.9 | 60.0 | 63.6 | 62.7 |
| James | 51.4 | 32.5 | 48.5 | 43.6 |

Note: UT = unconscious thought; CT = conscious thought; ID = immediate decision.

**Appendix D**

Cue definitions extracted from DePaulo et al. (2003):

1. Postural shifts: Postural adjustments, trunk movements, or repositionings of the body.
2. Facial pleasantness: Speaker’s face appears pleasant; speakers show more positive facial expressions (such as smiles) than negative expressions (such as frowns or sneers).
3. Fidgeting: Object fidgeting (speakers are touching or manipulating objects) and/or self-fidgeting (speakers are touching, rubbing, or scratching their body or face) and/or facial fidgeting (speakers are touching or rubbing their faces or playing with their hair).
4. Unfilled pauses: silent pauses; periods of silence.
